# A Lanosteryl Triterpene from *Protorhus longifolia* Improves Glucose Tolerance and Pancreatic Beta Cell Ultrastructure in Type 2 Diabetic Rats

**DOI:** 10.3390/molecules22081252

**Published:** 2017-07-26

**Authors:** Sihle E. Mabhida, Rebamang A. Mosa, Dambudzo Penduka, Foluso O. Osunsanmi, Phiwayinkosi V. Dludla, Tryana G. Djarova, Andy R. Opoku

**Affiliations:** 1 Department of Biochemistry and Microbiology, University of Zululand, KwaDlangezwa 3886, South Africa; sihlemabhida@gmail.com (S.E.M.); propafadzo@gmail.com (D.P.); alafin21@yahoo.com (F.O.O.); drdjarova@yahoo.com (T.G.D.); opokuA@unizulu.ac.za (A.R.O.); 2 Biomedical Research and innovation Platform (BRIP), South African Medical Research Council, Tygerberg 7505, South Africa; pdludla@mrc.ac.za

**Keywords:** type 2 diabetes, hyperglycemia, hyperlipidemia, oxidative stress, inflammation, pancreatic beta cells, antioxidants, triterpenes, *Protorhus longifolia*

## Abstract

Type 2 diabetes remains one of the leading causes of death worldwide. Persistent hyperglycemia within a diabetic state is implicated in the generation of oxidative stress and aggravated inflammation that is responsible for accelerated modification of pancreatic beta cell structure. Here we investigated whether a lanosteryl triterpene, methyl-3β-hydroxylanosta-9,24-dien-21-oate (RA-3), isolated from *Protorhus longifolia* can improve glucose tolerance and pancreatic beta cell ultrastructure by reducing oxidative stress and inflammation in high fat diet and streptozotocin-induced type 2 diabetes in rats. In addition to impaired glucose tolerance, the untreated diabetic rats showed increased fasting plasma glucose and C-peptide levels. These untreated diabetic rats further demonstrated raised cholesterol, interleukin-6 (IL-6), and lipid peroxidation levels as well as a destroyed beta cell ultrastructure. Treatment with RA-3 was as effective as metformin in improving glucose tolerance and antioxidant effect in the diabetic rats. Interestingly, RA-3 displayed a slightly more enhanced effect than metformin in reducing elevated IL-6 levels and in improving beta cell ultrastructure. Although the involved molecular mechanisms remain to be established, RA-3 demonstrates a strong potential to improve pancreatic beta cell ultrastructure by attenuating impaired glucose tolerance, reducing oxidative stress and inflammation.

## 1. Introduction

Incidence of type 2 diabetes mellitus, characterized by insulin resistance, is increasing at an alarming rate and remains a serious global health concern [1,2]. Lifestyle modifications such as excessive food intake and lack of physical activity are some of the factors contributing to cellular insulin insensitivity and subsequent insulin resistance [1,2]. Insulin resistance results in the abnormally elevated levels of circulating blood lipids “hyperlipidemia” and glucose “hyperglycemia” observed in type 2 diabetic patients. Hyperlipidemia and hyperglycemia are considered to be the main contributors to type 2 diabetes and associated complications [1,2]. Such complications include accelerated oxidative injury through enhanced generation of free radical species and inflammatory response [3,4,5]. Increased oxidative stress as well as aggravated inflammatory response in type 2 diabetes are widely-reported phenomena, and are known to cause cellular damage to various organs, including the pancreatic beta cells [3,4]. Furthermore, it has been reported that the increase in pro-inflammatory cytokines such as interleukine-1 (IL-1), tumor necrosis factor alpha (TNF-α) and interleukin 6 (IL-6) in a type 2 diabetic state is commonly accompanied by a decrease in cellular antioxidant levels and increased apoptosis [5].

**Figure 1:**
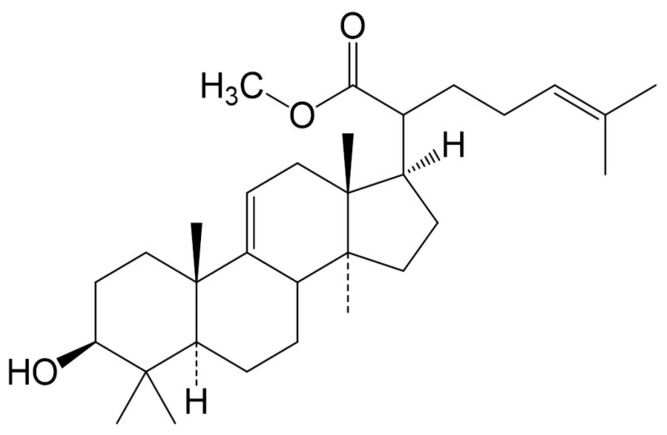
The chemical structure of methyl-3β-hydroxylanosta-9,24-dien-21-oate (RA-3).

The ideal approaches in the management of type 2 diabetes and its associated complications should focus on the development of drugs that would improve cellular insulin sensitivity and antioxidant levels. This would subsequently help to control blood glucose and lipid levels and protect pancreatic beta cells from oxidative damage [6,7]. The majority of synthetic antidiabetic drugs currently on the market have long-term side effects and some are single target [8]. Literature indicates that plant derived products seem to be multi-target and can be effective in either their crude and/or pure forms [9,10,11]. Triterpenes are a group of plant-derived bioactive compounds that continue to display a wide array of significant potential bioactivities [12,13]. These plant-derived compounds have been demonstrated to have important bioactivity to ameliorate hyperlipidemia [14,15], inflammation [16,17], and diabetes [13,18,19,20]. In addition, these compounds have potential in protecting and enhancing the regeneration of pancreatic islets cells [21], improving glucose tolerance [22] and other diabetic complications [13,18,19,20]. Recently, we have shown that a lanosteryl triterpene (Methyl-3β-hydroxylanosta-9,24-dien-21-oate; Figure 1) from the stem barks of *Protorhus longifolia* (Benrh.) Engl. (*Anacardiaceae*) possesses a broad spectrum of biological properties, including in vivo hypolipidemic and hypoglycemic properties [23,24,25,26,27]. We have already reported that RA-3 administration for 14 days suppressed glucose levels and increased hepatic glycogen content in streptozotocin (STZ)-induced diabetes in rats [20]. Thus, based on these results, we aimed to investigate whether longer RA-3 treatment can improve the ultrastructure of pancreatic beta cells through improving glucose tolerance in a high-fat diet and STZ-induced type 2 diabetes rat model.

## 2. Results

### 2.1. RA-3 Displayed Hypoglycemic Effect in the Type 2 Diabetic Rats

The biological effect of RA-3 on blood glucose and C-peptide levels of diabetic rats was determined and results are shown in Table 1. As opposed to markedly higher fasting plasma glucose (FPG) levels after the 28 days treatment period, C-peptide levels were significantly lower in the diabetic control group (29.0 ± 1.09, *p* ≤ 0.0001 and 0.2 ± 2.41, *p* ≤ 0.001) when compared to the non-diabetic controls (3.9 ± 0.04 and 0.8 ± 0.01, respectively). RA-3 treatment (4.3 ± 0.11, *p* ≤ 0.001 and 0.4 ± 0.14, *p* ≤ 0.05, respectively) showed a similar effect to metformin (4.5 ± 0.22, *p* ≤ 0.0001 and 0.4 ± 0.12, *p* ≤ 0.05, respectively) in reducing FPG and increasing C-peptide levels after the 28 days’ treatment period.

### 2.2. RA-3 Improved Glucose Tolerance in Type 2 Diabetic Rats

**Figure 2:**
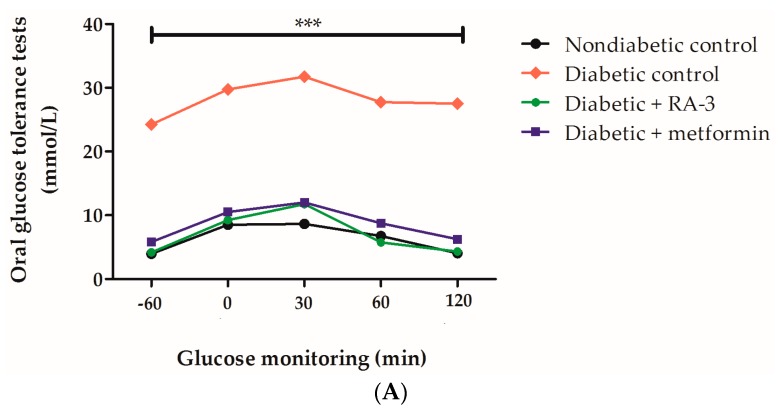

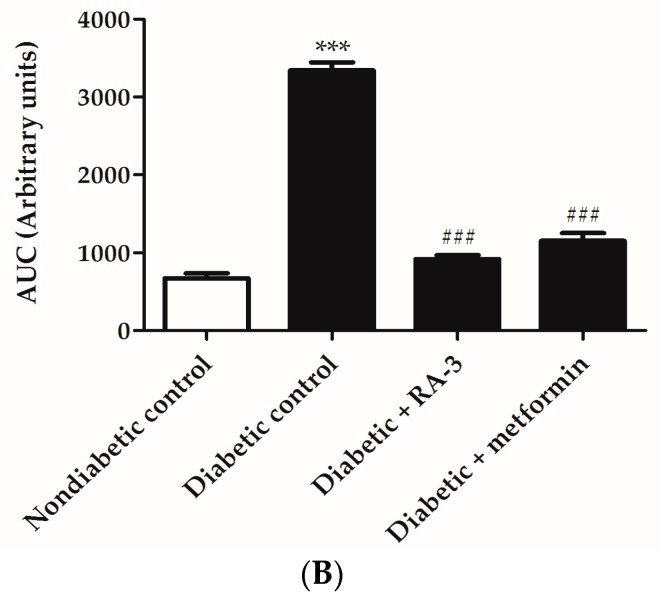
Oral glucose tolerance tests (**A**) and area under the curve (AUC) (**B**) in high fat diet and streptozotocin-induced type 2 diabetic rats treated with RA-3 and metformin (positive control). The untreated diabetic group presented with a significant increase in fasting plasma glucose levels (*** *p* ≤ 0.0001) compared to the non-diabetic rats and diabetic rats treated with RA-3 and metformin. ^###^ *p* ≤ 0.001 vs. diabetic control. Results are expressed as the mean ± SEM and each treatment group contained at least five rats. One way analysis of variance (ANOVA), followed by a Tukey post-hoc test (Graph Pad Prism version 5.03) were used to determine statistical differences. The values were considered statistically significant where *p* ≤ 0.05.

Non-diabetic and diabetic rats presented with increased levels of FPG levels from baseline (−60) to 30 min after administration of a 2 g/kg glucose bolus (Figure 2). However, these FPG levels were reduced in all animals after 30 min. Diabetic control animals displayed significantly elevated FPG levels (*p* ≤ 0.0001) when compared to either non-diabetic controls or the diabetic animals treated with RA-3 and metformin (Figure 2A). RA-3 was effective in reducing increased FPG in diabetic animals back to levels similar to those of the non-diabetic animals (Figure 2A) following the 28 days of the treatment period. Interestingly, the effect of RA-3 was similar to a commonly used antidiabetic drug, metformin. The improvement of oral glucose tolerance with RA-3 and metformin treatment was confirmed by ‘area under the curve’ results (Figure 2B).

### 2.3. RA-3 Prevented Lipid Peroxidation through Enhancement of Endogenous Antioxidant Status in the Type 2 Diabetic Rats

The increased malondialdehyde (MDA) levels, as an indication of lipid peroxidation, were significantly higher in the diabetic control group (1.31 ± 0.008, *p* ≤ 0.0001) than the non-diabetic control (0.37 ± 0.004) (Table 2). Similarly, antioxidant markers such as glutathione (GSH), superoxide dismutase (SOD) and catalase (CAT) were markedly reduced in the diabetic control group (2.38 ± 0.01, *p* ≤ 0.0001; 30 ± 0.012, *p*≤ 0.001; and 0.08 ± 0.004, *p* ≤ 0.05, respectively) when compared to the non-diabetic control (7.33 ± 0.01, 56 ± 0.005, 0.12 ± 0.005, respectively) (Table 2).Treatment with RA-3 presented a comparable effect to metformin in enhancing GSH content (4.40 ± 0.006, *p* ≤ 0.05 and 4.10 ± 0.003, respectively), CAT (0.13 ± 0.001, *p* ≤ 0.05 and 0.11 ± 0.005, *p* ≤ 0.05, respectively) and SOD (41 ± 0.004 and 39 ± 0.004, respectively) activity, though non-significantly for the latter.

### 2.4. RA-3 Reduced Cholesterol and Interleukin-6 Levels While it Improved the Pancreatic Beta Cell Ultrastructure of the Type 2 Diabetic Rats

**Figure 3:**
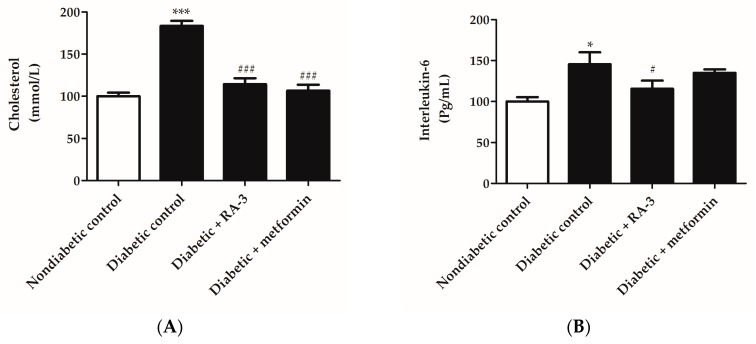
The effect of RA-3 on (**A**) plasma cholesterol and (**B**) serum interleukin-6 (IL-6) levels in the high fat diet and streptozotocin-induced type 2 diabetic rats. Results are expressed as the mean ± SEM and each treatment group contained at least five rats. * *p* ≤ 0.05, *** *p* ≤ 0.0001 vs. non-diabetic control, ^#^ *p* ≤ 0.05, ^###^ *p* ≤ 0.001 vs. diabetic control. One way analysis of variance (ANOVA), followed by a Tukey post-hoc test (Graph Pad Prism version 5.03) were used to determine statistical differences. The values were considered statistically significant where *p* ≤ 0.05.

**Figure 4:**
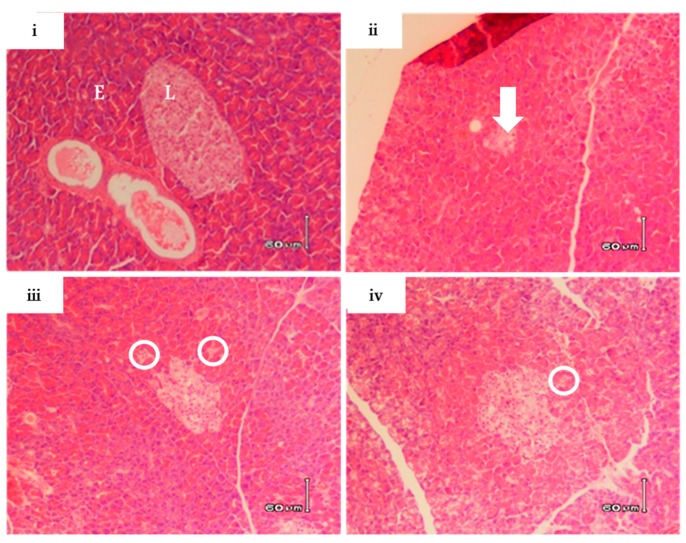
The effect of RA-3 on pancreatic beta cell ultrastructure in the high fat diet and streptozotocin-induced type 2 diabetic rats. (**i**) Normal structure of pancreatic islets from non-diabetic rats; (**ii**) A destructed structure as well as a reduced size of a pancreatic islets in untreated diabetic rats; (**iii**) An improved structure of pancreatic islets with some debris in diabetic rats treated with RA-3; (**iv**) A reduced size of pancreatic islets with debris of cells in diabetic rats treated with metformin. (L)-designates a normal structure of islets of Langerhans, (E) exocrine portion of pancreatic tissue, and an arrow shows destructed and condensed endocrine cells while a circle illustrates debris of destructed cells. NB: The indicator size for each image is 60 µm.

The diabetic control group displayed significantly increased plasma cholesterol (183.4 ± 5.92, *p* ≤ 0.0001) and serum IL-6(145.5 ± 14.55, *p* ≤ 0.05) levels in comparison to the non-diabetic rats (100.0 ± 4.21 and 100.0 ± 5.4, respectively) (Figure 3A,B). In addition, haematoxylin and eosin (H&E) stain demonstrated a condensed pancreatic beta cell ultrastructure in the diabetic control group (Figure 4). However, treatment with RA-3 was as effective as metformin in reducing plasma cholesterol levels (114.2 ± 7.2, *p* ≤ 0.0001 and 106.5 ± 7.22, *p* ≤ 0.0001, respectively) (Figure 3A), while it displayed a better effect than metformin in reducing IL-6 levels (115.5 ± 10.1, *p* ≤ 0.05 and 135.0 ± 4.3, respectively) (Figure 3B). Interestingly, RA-3 treatment improved the pancreatic beta cell ultrastructure in comparison to the diabetic control rats and diabetic rats treated with metformin (Figure 4).

## 3. Discussion

Type 2 diabetes constitutes almost 90% of diabetes mellitus, and remains one of the leading causes of death worldwide [1,2]. Although commonly used antidiabetic and antilipidemic drugs such as metformin and atorvastatin prolong the lives of diabetic patients, the estimated rate of deaths due to diabetes continues to increase each year [1,2]. This has resulted in an increased exploration of natural products not just to lower elevated FPG levels but to improve the structure and function of pancreatic beta cells [21,28]. It is increasingly reported that persistent hyperglycemia through accelerated oxidative stress and inflammation may contribute to the loss of beta cell function [3,7]. Here we investigated the effect of RA-3 on improving glucose tolerance as well as associated markers of oxidative stress and inflammation in correlation with pancreatic beta cell ultrastructure in the high fat diet and STZ-induced type 2 diabetic rats.

A high fat diet and STZ-induced diabetic rat model is a well characterized system to study complications associated with type 2 diabetes [12,23]. In the present study, the type 2 diabetic rats displayed impaired glucose tolerance and hyperlipidemia which was evidenced by increased plasma cholesterol levels. This was concomitant to reduced serum C-peptide levels as an indication of low productivity of insulin by the beta cells. These rats further presented with reduced serum antioxidants, increased lipid peroxidation end product, MDA, and inflammatory marker, IL-6, while damage to the structure of pancreatic beta cells was also evident. It is hypothesized that hyperglycemia and fluctuations in blood glucose levels in the diabetic state result in excessive production of free radical species, which may lead to oxidative stress [4,29]. This complication is implicated in the progression of long-term diabetes consequences including damage to pancreatic beta cells [3,21,28]. Once hyperglycemia has developed, inflammation has been another factor linked to glucotoxicity and accelerated beta cell destruction, leading to phenotypic alterations and loss of beta cell mass through apoptosis [3,21,28]. Therefore, optimal islet beta cell function is vital for efficient insulin release and subsequent improved cellular glucose uptake.

The lanosteryl triterpene, RA-3, first identified from the stem bark of *Protorhus longifolia* [27] has already been established as containing hypolipidemic properties by reducing total serum cholesterol and low density lipoprotein cholesterol in vivo [23], while its hypoglycemic potential was demonstrated in STZ-induced diabetic rats [20]. Results obtained from this study clearly demonstrated that RA-3 exerts a similar effect to that of metformin in improving glucose tolerance as well as FPG, cholesterol and C-peptide levels in the type 2 diabetic rats. In addition, RA-3 was more effective than metformin in reducing IL-6 levels. This is an interesting result since cytokines such as IL-6 are considered as the main regulators of inflammation during diabetic pathogenesis [30], and only a few compounds have displayed similar or better ameliorative effect of reducing diabetes associated complications than metformin. Supporting these results, Dudhgaonkar and colleagues have previously demonstrated that a triterpene extract from *Ganoderma lucidum* suppressed the secretion of IL-6 in lipopolysaccharide-stimulated macrophages [31]. A recent study showed that a novel triterpene (2α, 3β, 19α-trihydroxy-24-oxo-olean-12-en-28-oic acid), isolated from Chinese acorns (*Quercusserrata var. brevipetiolata*) can inhibit tumor necrosis alpha (TNF-α)-induced IL-6 and IL-8 production in MH7A cells [17]. Although further studies are required to assess the synergistic use of RA-3 and metformin, the results obtained from this study support recent evidence promoting the combined use of natural products with current therapies to reduce the burden of type 2 diabetes and associated complications.

Furthermore, accumulating interest in the use of natural compounds such as RA-3 has been attributed to their strong antioxidant properties [20,24], which are essential in the prevention of hyperglycemia-induced inflammation and cellular damage. In agreement with previous findings [24], this study demonstrated that RA-3 can suppress lipid peroxidation, by reducing MDA levels, whereas this effect was parallel to raised antioxidant levels as measured by the assessment of serum GSH and CAT levels. GSH is one of the most important and abundant antioxidants in the body, while CAT remains essential in the detoxification of highly reactive hydrogen peroxide [29]. Antioxidant properties of RA-3 were consistent with the effect of improving beta cell ultrastructure in type 2 diabetic rats. Experimental data has already been presented that natural compounds such as resveratrol, a stilbenoid found in abundance in the skin of grapes and red wine, and aspalathin, a dihydrochalcone *C*-glucoside unique to *Aspalathus linearis,* present similar effects in reducing oxidative damage by preventing oxidative stress and inflammation in a diabetic state [29,32,33]. Moreover, these compounds, through their robust antioxidant effects, can improve the ultrastructure of the pancreatic beta cells and myocardium of type 2 diabetic mice [29,32,33]. The molecular mechanisms associated with the protective effect of resveratrol and aspalathin have been associated with modulation of intracellular energy homeostasis or antioxidant response through 5′ adenosine monophosphate-activated protein kinase (AMPK) and nuclear factor (erythroid-derived 2)-like 2 (Nrf2), respectively [29,32,33]. Oleanolic acid, a naturally occurring pentacyclic triterpenoid, has been previously demonstrated to protect mice against acetaminophen hepatotoxicity through the activation of Nrf2 and its downstream target genes including those involved in GSH synthesis [34]. Indeed, increased levels of antioxidants such as GSH and CAT as well as other inflammatory markers have been shown to be mainly mediated by Nrf2 activation in various disease models [34,35,36]. Thus, although additional studies are required, the strong antioxidant effects of RA-3 to combat diabetes-associated complications as well as improving altered pancreatic beta cell ultrastructure could be attributed to its capacity to upregulate Nrf2 expression.

In summary, results obtained from this study demonstrate that RA-3 improved glucose tolerance and pancreatic beta cell ultrastructure by inhibiting inflammation through the reduction of IL-6 levels and enhanced antioxidant status of the type 2 diabetic rats. The potential molecular mechanism by which RA-3 improves the ultrastructure of beta cells remains to be elucidated. However, recent research has highlighted that, similar to oleanolic acid, resveratrol and aspalathin, RA-3 may potentially induce its effect by modulating AMPK or Nrf2 [34,35,37]. Thus, future research directions, which are also important in addressing limitations of the current study, involve unravelling molecular mechanisms associated with the protective effect of RA-3, including its effect on regulating pancreatic beta cell function as well as insulin secretion in isolated islet and plasma insulin levels. These investigations will take into account both RA-3 as a monotherapy or in combination with metformin.

## 4. Materials and Methods

### 4.1. Reagents 

Unless otherwise specified, all reagents, chemicals and assay kits used were from Sigma-Aldrich Chemical Co. (St. Louis, MO, USA).

### 4.2. Extraction and Compound Isolation

*Protorhus longifolia* fresh stem bark (specimen voucher number RAUZ01) was harvested from the KwaHlabisa area in KwaZulu-Natal, South Africa. The plant material was cleaned and routinely prepared for extraction. The triterpene was extracted and isolated from the powered *Protorhus longifolia* stem bark as previously reported [27]. Briefly, *n*-hexane was used to defat (1:5 *w*/*v*) the powdered plant material and the defatted plant material was extracted with chloroform. The targeted lanosteryl triterpene (RA-3) was then isolated from the chloroform extract using silica gel chromatography (silica gel 60; 70–230 mesh ASTM; 0.063–0.2 mm; Merck, Billerica, MA, USA). The column was step-wisely eluted with an *n*-hexane:ethyl acetate solvent system. The collected small fractions (20 mL) were analyzed with thin layer chromatography. RA-3 was obtained following its recrystallization in ethyl acetate (100%). The chemical structure of the compound (Figure 1) was confirmed using spectral techniques. The obtained physical and spectral data were in agreement with previous reports [27].

### 4.3. Animals

Ethical clearance (UZREC 171110–030 PGM 2016/329) for approval of procedures and use of laboratory animals was obtained from the University of Zululand Research Ethics Committee (UZREC). Sprague-Dawley rats (150–200 g) were obtained from the laboratory animal unit of Biochemistry and Microbiology Department, University of Zululand. The animals were maintained under standard conditions, in a controlled environment with a 12 h light/dark cycle in a temperature range of 23–25 °C (relative humidity ~50%), as outlined in the institutional and national guidelines for handling and caring of science laboratory animals. Animals were allowed five days acclimatization period with free access to enough normal rat feed and drinking water before subsequent experiments.

### 4.4. Establishment of a Type 2 Diabetic Rat Model

The method described by Machaba et al. [23] was followed with some modification to induce hyperlipidemia in rats. The Sprague Dawley rats of either sex were put on a high fat diet (pellets containing: commercial rat chow (79.3%), sunflower oil (15%), bile salt (0.5%), cholesterol (5%), and Thirmecil (0.2%) for a period of 28 days. The rats in the control group were fed a standard rodent diet. Hyperlipidemic condition in the animals was confirmed by measuring blood cholesterol levels (Accutrend cholesterol meter; Roche Diagnostics, Mannheim, Germany) from the rat’s tail tip. Animals with the blood cholesterol level equal to or above 5.2 mmol/L were considered hyperlipidemic and used in the study.

After 28 days on high fat diet, the hyperlipidemic rats were fasted overnight. Thereafter, the rats were given intraperitoneal injection of a low single dose of a freshly prepared STZ solution (30 mg/kg). After five days of the STZ injection, blood glucose levels were measured from the blood collected from the tail tip with a glucometer (Accutrend glucometer; Roche Diagnostics, Mannheim, Germany). Animals with blood glucose levels equal to or above 11 mmol/L were considered diabetic and used in the study.

### 4.5. Treatment of High Fat Diet- Induced Diabetic Rats with RA-3

The diabetic rats were randomly divided into five groups of at least five rats per group. The rats in the experimental group received a daily single oral dose of 100 mg/kg of either RA-3 or metformin, a known antidiabetic drug, for 28 days. Animals in the non-diabetic and diabetic control groups received a daily single oral administration of distilled water and 2% Tween 20 (vehicle), respectively. RA-3 and metformin were dissolved in 2% Tween 20 and distilled water, respectively, before orally administered to the rats at the same time (08:00–09:00), and the dose used was based on a previously published study [20].

### 4.6. Oral Glucose Tolerance Test

At the end of the 28 days treatment period, the animals were fasted for 12 h and then received an oral glucose load (2 g/kg body weight). Changes in postprandial blood glucose levels were then monitored at baseline (−60), 0, 30, 60, 120 minute intervals. No visible side effects were observed in animals after the treatment period.

### 4.7. Determination of Fasting Plasma Glucose Levels

In rats fasted overnight, fasting plasma glucose levels were measured by tail prick using a handheld glucometer (Accutrend glucometer, Roche Diagnostics, Mannheim, Germany).

### 4.8. Biochemical Analysis

A day after oral glucose tolerance tests, rats were fasted for 4 hours before being weighed and anesthetized. Animals received the anesthetic until no reaction could be recorded by pedal reflex before removal of blood. Blood was centrifuged at 4000 g at 4 °C for 15 min before the serum was removed for analysis of the levels of C-peptide, MDA, GSH, SOD and CAT using respective commercial assay kits (Sigma-Aldrich, St. Louis, MO, USA), as per manufacturer’s instructions. IL-6 was assayed using an enzyme-linked immunosorbent assay (ELISA) kit (Sigma-Aldrich, Steinheim, Germany).

### 4.9. Histopathological Studies

Following anesthezia and confirmation of no pedal reflex in the animals, pancreatic tissue was excised and preserved in 10% (*v*/*v*) neutral buffered formalin for histological studies. Tissue slides were prepared following standard procedures. Hematoxylin and eosin (H&E) were used to stain pancreas tissues for histopathological analysis by photomicroscope (Vet Diagnostix Laboratories, Pietermaritzburg, South Africa). The slide examination was performed by a qualified pathologist with no prior knowledge of the respective animal groups.

### 4.10. Data Analysis

The experiments were replicated at least three times and reported as the mean ± standard error of mean (S.E.M). One way analysis of variance (ANOVA), followed by a Tukey post-hoc test or unpaired Student *t*-test where appropriate (Graph Pad Prism version 5.03) were used to determine statistical differences. The values were considered statistically significant where *p* ≤ 0.05.

## Figures and Tables

**Table 1:** The effect of RA-3 on fasting plasma glucose (FPG) and C-peptide levels after the 28 days treatment of the high fat diet and streptozotocin-induced type 2 diabetic rats.

Experimental Group	FPG Day 0 (mmol/L)	FPG Day 28 (mmol/L)	C-peptide Day 28 (µg/L)
Non-diabetic control	4.1 ± 0.22	3.9 ± 0.04	0.8 ± 0.01
Diabetic control	18.4 ± 0.78 ***	29.0 ± 1.09 ***	0.2 ± 2.41 ***
Diabetic + RA-3	11.5 ± 0.38 ***^,###^	4.3 ± 0.11 ^###^	0.4 ± 0.14 *^,#^
Diabetic + metformin	15.7 ± 0.66 ***^,#^	4.5 ± 0.22 ^###^	0.4 ± 0.12 *^,#^

Day 0 was included to show that rats were already diabetic by the commencement of treatment (FPG ≥ 11 mmol/L). Results are expressed as the mean ± SEM and each treatment group contained at least five rats. * *p* ≤ 0.05, *** *p* ≤ 0.0001 vs. non-diabetic control, ^#^ *p* ≤ 0.05, ^###^ *p* ≤ 0.0001 vs. diabetic control. One way analysis of variance (ANOVA), followed by a Tukey post-hoc test (Graph Pad Prism version 5.03) were used to determine statistical differences. The values were considered statistically significant where *p* ≤ 0.05.

**Table 2:** The effect of RA-3 on lipid peroxidation and antioxidant levels after the 28 days treatment of the high fat diet and streptozotocin-induced type 2 diabetic rats.

Experimental Group	GSH Content (nmol/mL)	SOD Activity (Inhibition Rate %)	CAT Activity (Units/mL)	MDA Levels (nmol/µL)
Nondiabetic control	7.33 ± 0.01	56 ± 0.005	0.12 ± 0.005	0.37 ± 0.004
Diabetic control	2.38 ± 0.01 ***	30 ± 0.012 **	0.08 ± 0.004 *	1.31 ± 0.008 ***
Diabetic + RA-3	4.40 ± 0.006 *^,#^	41 ± 0.004 *	0.13 ± 0.001 ^#^	0.75 ± 0.005 *^,##^
Diabetic + metformin	4.10 ± 0.003 *^,#^	39 ± 0.004 *	0.11 ± 0.005 ^#^	0.53 ± 0.003 *^,##^

Results are expressed as the mean ± SEM and each treatment group contained at least five rats. * *p* ≤ 0.05, ** *p* ≤ 0.001, *** *p* ≤ 0.0001 vs. non-diabetic control, ^#^ *p* ≤ 0.05, ^##^ *p* ≤ 0.001 vs. diabetic control. CAT: catalase, GSH: glutathione, MDA: malonaldehyde. One way analysis of variance (ANOVA), followed by an unpaired Student *t*-test (Graph Pad Prism version 5.03) were used to determine statistical differences. The values were considered statistically significant where *p* ≤ 0.05.
